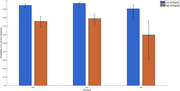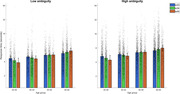# Perceptual Discrimination of Complex Objects: Gene‐dose effects of APOE e4 in mid‐life

**DOI:** 10.1002/alz.095233

**Published:** 2025-01-09

**Authors:** Claire L Lancaster, Sam Berens, Jessica Daly, Jennifer M Rusted, Chris Bird

**Affiliations:** ^1^ Brighton & Sussex Medical School, Brighton United Kingdom; ^2^ University of Sussex, Brighton United Kingdom

## Abstract

**Background:**

The perirhinal cortex is vulnerable to early phosphorylated tau (p‐tau) accumulation; deterioration of this region in the prodromal stages of Alzheimer’s Disease (AD) is associated with impaired ‘complex’ perceptual discrimination. This research examined whether there is a disadvantageous gene‐dose effect of Apolipoprotein (APOE) e4 on perceptual discrimination in mid‐life, facilitating greater understanding of how and when the deleterious effects of this variant emerge.

**Methods:**

Three‐hundred and thirteen mid‐aged adults (45‐65 years; 51% female; recruited by NIHR BioResource) completed a Greebles ‘odd‐one‐out’ task. Participants were grouped by APOE e4 gene‐dose (142 APOE33 controls, 135 APOE34, 36 APOE44). Frequentist and Bayesian Generalised Linear Mixed Effect Models probed genotype differences in probability and speed of correct perceptual discrimination, including in interaction with age.

**Results:**

A significant non‐linear effect of APOE e4 gene dose was reported on probability of correct perceptual discrimination (*F* = 3.75, *p* = .25, Bayesian 95% Credibility Intervals (CI) = [.07, 1.90], driven by APOE44 performing worse than APOE33 controls and APOE34 carriers (Figure 1). In addition, there was a significant Age x APOE e4 gene‐dose interaction (*F* = 9.75, *p* = .002, Bayesian 95% CIs = [.00, .61]), with the slope of age‐related slowing increasing as a function of the number of e4 alleles carried (Figure 2). Estimates suggest high‐risk individuals are quicker than the APOE33 control group until age 54, but slower after age 60.

**Conclusions:**

Observed disadvantageous effects of APOE e4 on a perirhinal‐dependent perceptual discrimination task may indicate this region is prematurely compromised in carriers of this genetic risk variant, potentially through its influence on rate of p‐tau aggregation. These results highlight the utility of perceptual discrimination tasks as a sensitive, scalable marker of increased risk for neurodegenerative disease.